# Dietary inflammatory index in relation to severe coronary artery disease in Iranian adults

**DOI:** 10.3389/fnut.2023.1226380

**Published:** 2023-09-29

**Authors:** Zahra Dadaei, Mohammad Bagherniya, Omid Sadeghi, Alireza Khosravi, Shahin Shirani, Gholamreza Askari

**Affiliations:** ^1^Nutrition and Food Security Research Center, Department of Community Nutrition, School of Nutrition and Food Science, Isfahan University of Medical Sciences, Isfahan, Iran; ^2^Student Research Committee, Isfahan University of Medical Sciences, Isfahan, Iran; ^3^Department of Community of Cardiology, Isfahan Cardiovascular Research Institute, Isfahan University of Medical Sciences, Isfahan, Iran; ^4^Hypertension Research Center, Cardiovascular Research Institute, Isfahan University of Medical Sciences, Isfahan, Iran

**Keywords:** the financial support for conception, design, data analysis, dietary inflammation index, severe CAD, gensini scoring system

## Abstract

**Background:**

Limited findings are available on the relationship between dietary inflammation index (DII) and severe coronary artery disease (CAD). Considering the high prevalence of CAD and its complications, we examined the relationship between DII and CAD.

**Methods:**

This cross-sectional study was conducted on 275 adults who underwent elective angiography. Severe coronary artery disease was measured by the gensini scoring system. DII was measured by a valid semi-quantitative 168-item food frequency questionnaire (FFQ). Blood samples were collected after 12 h of fasting to measure serum lipid profile and quantitative C-reactive protein (q-CRP) levels. Binary logistic regression was used to calculate the odds (OR) and 95% confidence interval (CI).

**Results:**

People in the last tertile of the DII had a higher chance of suffering from severe coronary artery disease (OR: 3.71; 95% CI: 1.97–6.98), hypercholesterolemia (OR: 2.73; 95% CI: 5.03–1.48), reduced HDL-cholesterol levels (OR: 3.77; 95% CI: 9.34–1.52), and hypertension (OR: 1.93; 95% CI: 3.49–1.06) compared to people in the first tertile. After adjusting for confounding factors, the relationship remained significant. A direct and significant relationship was observed between the DII and increased q-CRP levels, which disappeared after adjusting for confounding factors in the adjusted model (OR: 2.02; 95% CI: 0.86–4.73).

**Conclusion:**

This cross-sectional study showed a direct and linear relationship between following an anti-inflammatory diet and decreasing the chance of severe CAD. Therefore, it seems necessary to implement community-based educational programs to promote healthy nutrition in order to prevent CADs.

## Introduction

Heart diseases and cardiovascular diseases (CVDs) are the most important cause of death in industrialized and developing countries (1). The prevalence of CVDs is increasing leading to mortality and reduced quality of life from childhood to old age (2). The World Health Organization reported that about 17.3 million deaths in 2008 were due to CVDs (30% of all deaths) and it is estimated that by 2030, there will be about 23.6 million deaths due to heart diseases, especially stroke (3). Coronary artery disease (CAD) is the main cause of death and disability in the population of Iran and accounts for approximately 50% of deaths each year (4).

CAD is characterized by atherosclerosis in epicardial coronary arteries (5). The angiographic severity is important in the progression and prognosis of CAD, and gensini scoring is more reliable compared to other methods of grading its severity. In addition, gensini scoring provides a quantitative variable compared to other systems, which is more valid in statistical analyses (6, 7). Age, gender, and family history are unchangeable risk factors, and tobacco use, diabetes, lack of physical activity, unhealthy diet, and stress are modifiable CAD risk factors (5). Diet plays an important role in regulating chronic inflammation, lipid, and blood pressure dysregulation, and increasing the risk of CVDs (8–13). The dietary inflammatory index (DII) is a dietary index designed by South Carolina University researchers to measure the inflammatory potential of diet (14). In previous studies, the DII score obtained from the food frequency questionnaire (FFQ) was significantly related to inflammatory biomarkers so that higher DII scores (indicating a diet causing more inflammation) showed a direct relationship with interleukin-6 (IL-6), tumor necrosis factor receptor 2 alpha (TNFα-R2), and C-reactive protein (CRP) levels (15).

Eating healthy diets reduces the risk of developing CAD (16, 17). Also, lowest adherence to an anti-inflammatory diet and the risk of CVDs are associate (18, 19). However, it has not been confirmed in some studies and different findings have been reported in men and women (20, 21). No study has investigated the relationship between DII and severe CAD using the gensini score in IRAN; therefore, considering the high prevalence of CVD and related costs imposed on societies, it is of considerable importance to provide new strategies to prevent the disease and find effective treatments with fewer complications. The purpose of this study was to investigate the relationship between DII and severe CAD in adults. Accordingly, by understanding the dietary patterns of these patients, we can provide practical recommendations and take a step toward the health of society by promoting the correct dietary pattern.

## Materials and methods

### Study design and participants

The current cross-sectional observational study was conducted on adults of both sexes as the target population in 2021. The sample size was calculated to be 217 people based on the formula for confidence interval of 95%, precision (d) of 10 and 63.8% prevalence of sever CAD based on similar articles (17), Since covid-19 pandemic was very prevalent during our data collection and the possibility of drop-out was high, we invited a total of 275 individuals, rather than 217 subjects, to participate in the study.

The subjects were selected from the patients admitted to the Elective Angiography Department of Shahid Chamran and Asgaria Hospital in Isfahan, one of the big central cities of Iran, aged 25 to 75 years and underwent diagnostic coronary angiography and were willing and able to participate in the study.

**Figure 1:**
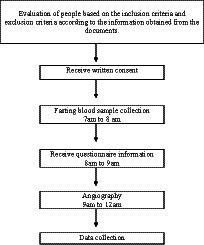
Process and timeline of the study design.

However, those following the exclusion criteria were excluded: (1) using supplements and anti-inflammatory drugs, (2) smoking and alcohol use, (3) following a special diet, a history of cancer, heart failure, heart attack, percutaneous coronary intervention (PCI), and coronary artery bypass grafting (CABG), stage 3 or higher chronic kidney disease, specific liver disease or receiving medication for liver disorders, immune system impairment, and AIDS, and (4) those with restrictions on receiving food by mouth for any reason. Written informed consent was obtained from all participants. Process and timeline of the study design is shown in Figure 1. The study protocol was approved by the Ethics Committee of Isfahan University of Medical Sciences (IR.MUI.RESEARCH.REC.1399.376).

### Assessment of dietary intakes

Food intakes were evaluated using a semi-quantitative 168-item food questionnaire (FFQ) specifically designed and validated for Iranian adults (22). According to a previous study on its validity, the dietary intake of 132 middle-aged adults using FFQ was assessed in comparison to a 24-h dietary recall (24HR). The correlation coefficients between food intake obtained from FFQ and 24HR were 0.59 for fat, 0.55 for total energy intake, 0.65 for proteins, 0.65 for magnesium, and 0.67 for fiber. The reliability of the FFQ was also evaluated by comparing the consumption of nutrients obtained from the FFQ at two time points with an interval of 1 year. In general, this FFQ has reported a valid and reliable tool for evaluating the common dietary intakes in Iranian adults (22). The FFQ was completed by a senior nutritionist with a face-to-face interview and the frequency and amount of food consumed by the participants in the last year were reported. Then, using household criteria, the amount of consumed foods was converted into grams per day (23). Finally, all the food items were transferred to the Nutritionist IV (N4) software, and the daily consumption of energy and all the nutrients were calculated.

### Evaluation of dietary inflammation index

The amounts of micronutrients to calculate the DII score were initially obtained as the mean and standard deviation of each food item. The z-score was obtained by subtracting the international standard average from the value derived from the FFQ and dividing it by the standard deviation. The z-score was then converted to a centered percentile score. The centered percentile score of each food item for each person was multiplied by the corresponding effect score of the food items (inflammatory potential for each food item) to obtain the DII score, and then by summing the score of food items for each person, the overall DII score was calculated (8). The nutritional items included energy, protein, total fat, Monounsaturated fatty acids (MUFA), polyunsaturated fatty acids(PUFAs), saturated fatty acids (SFAs), omega-6 fatty acids containing multiple double bonds, omega-3 fatty acids with multiple double bonds, trans fatty acid, cholesterol, carbohydrate, fiber, caffeine, vitamin A, beta-carotene, thiamine, riboflavin, niacin, vitamin B6, folate, vitamin B12, vitamin C, vitamin D, vitamin E, iron, magnesium, selenium, zinc, tea, garlic, onion, saffron, turmeric, ginger, pepper, thyme, rosemary, flavones, flavone 3l, flavonols, isoflavones, flavanones, anthocyanins, alcohol, and eugenol. A higher score reflects a diet with a higher degree of inflammation and vice versa (8). According to the items of the questionnaires and software used in this study, the DII with 32 items out of 45 reference items (except trans fatty acid, rosemary, saffron, ginger, thyme, flavones, flavone 3l, flavonols, isoflavones, flavanones, anthocyanins, alcohol, and eugenol) was calculated.

### Evaluation of gensini score

Gensini score was calculated as mentioned earlier (24, 25). Those with a gensini score of 20 or more were considered to have severe coronary artery disease, which is roughly equivalent to a 70% or more blockage of the left anterior descending (LAD) artery (26, 27).

### Assessment of biochemical markers

To evaluate the levels of blood lipids and q-CRP, 5 cc of fasting blood samples (12 h) were taken from the subjects. The blood samples were centrifuged for 10 min at 3000 rpm and the resulting serum was stored in a freezer at −20°C. Triglyceride (TG) and high-density lipoprotein cholesterol (HDL-C) concentrations were measured by enzymatic colorimetric method and total cholesterol concentration was also measured by a photometric method using French Cobus autoanalyzer (Pars Azmoun kit, Tehran, Iran). The concentration of LDL-C was also calculated using the Friedewald formula [LDL = TC – HDL – 1.5 (TG)] (28). The optimum value for total cholesterol was <200 mg/dl, for HDL was >40 mg/dl in men and > 50 mg/dl in women, and for LDL-C was <100 mg/dl and for TG < 150 mg/dl (29).

The serum level of q-CRP was measured quantitatively by the immunoturbidimetric method using the laboratory kit (Byrex Fox, Fars, Iran) with a cutoff point of 10 mg/L (30).

### Assessment of other variables

Blood pressure (BP) was measured using a digital sphygmomanometer (OMRON, M3, HEM-7154-E, Japan) with an accuracy of 0.5 mmHg, twice for each participant after 5 min of resting time in a sitting position and their average was recorded (31). High blood pressure is considered as the average systolic blood pressure ≥ 130 mm Hg or the average diastolic blood pressure ≥ 80 mm Hg (31).

Anthropometric indicators, including weight (with light clothes and without shoes using a body composition analyzer (Tanita MC-780MA, Tokyo, Japan), with an accuracy of 0.1 kg) and height (without shoes using a non-elastic meter mounted on the wall) were measured. Body mass index (BMI/kg/m^2^) was also calculated by dividing weight (kg) by the square of height (in meters).

Physical activity was evaluated using the International Valid Physical Activity Questionnaire (IPAQ) (32), which its validity and reliability have been measured in Iran (33). Demographic, socioeconomic characteristics, confounding and contextual variables, such as age, gender, education level, medical history, drug intake, and supplement use, were obtained using a general information questionnaire.

### Statistical methods

The normal distribution of the variables was investigated using the Kolmogorov–Smirnov test. The values of quantitative and qualitative variables were presented as mean (± standard deviation) and percentage, respectively. First, subjects were ranked based on DII (energy-adjusted) tertiles. The chi-square test was used to compare qualitative variables and one-way analysis of variance (ANOVA) was used to compare quantitative variables in DII tertiles. Also, energy-adjusted dietary intakes of participants across tertiles of DII were evaluated by one-way analysis of variance (ANOVA). Binary logistic regression was used to report the odds ratio (OR) and 95% confidence interval (CI) for severe CAD, lipid profile, BP, and q-CRP in different DII tertiles in crude and adjusted models. In the adjusted model, age, sex, BMI, physical activity, medication use, medical history, number of family members, and education were adjusted. The first quartile of DII was considered the reference group in the crude and adjusted model. DII tertiles were considered as continuous variables to determine the P trend in binary logistic regression models. In addition, the raw and adjusted values (energy intake, age, sex, BMI, physical activity, taking medication, medical history, number of family members, and education) average gensini score, lipid profile values, q-CRP levels, and BP in DII tertiles was reported using ANCOVA. Statistical analyses were performed using SPSS 26 (SPSS Inc., version 0.21, Chicago, IL). *p*-values less than 0.05 were considered statistically significant.

## Results

In this cross-sectional study, 275 Iranian adults referring to Chamran and Asgaria hospitals (a government hospital and a private hospital) for angiography were studied, of whom 59.3% were men. The average age, weight, and BMI of the participants were 59.10 ± 8.57 years, 77.63 ± 11.17 kg, and 28.5 ± 4.06 kg/m^2^, respectively. Also, 59.6% of people had severe CAD and the average DII was −0.50 ± 4.49. The general characteristics of the participants regarding DII tertiles are presented in Table 1. Those in the upper tertiles of the DII were older and more anticoagulant drug consumption, had higher average weight and higher economic status, and were found with less fatty liver and diabetes compared to the lower tertiles. There was no significant difference in the distribution of other variables among the tertiles of the DII. The food intake of the participants in the study is presented in Table 2. Those in the upper tertile of the dietary inflammatory index had less intake of nuts, whole grains, carbohydrates, thiamin, vitamin D, pepper and tea than the lower tertiles. Also, those in the upper tertile of the DII were found with higher intakes of energy, protein, fat, SFA, monounsaturated fatty acid (MUFA), polyunsaturated fatty acid (PUFA), cholesterol, omega-3, omega-6, iron, zinc, vitamin B2, vitamin B6, vitamin B9, vitamin B12, vitamin C, vitamin E, vitamin A, beta carotene, onions, legumes, refined grains, red and processed meat, and vegetables compared to than the lower tertiles. No other significant difference in dietary intakes was observed among the tertiles of the dietary inflammatory index.

**Table 1:** General characteristics of study participants across tertile of DII (energy-adjusted).^1^

	T1 (*n* = 91)	T2 (*n* = 92)	T3 (*n* = 92)	*p*-value^2^
Demographic variables				
Sex, (Male) (%)	56 (61.5)	54 (58.7)	53 (57.6)	0.85
Age (year)	57.26 ± 7.28	59.29 ± 9.45	60.69 ± 8.09^a^	0.02
Weight (kg)	73.54 ± 11.59	78.85 ± 10.25	80.44 ± 10.55^a^	<0.001
BMI^3^ (kg/m^2^)	27.25 ± 4.59	29.00 ± 3.65	29.16 ± 3.63^a^	0.001
Physical activity (MET. min/wk)	1311.97 ± 360.40	1250.32 ± 387.28	1253.07 ± 369	0.45
Education^3^ (University graduated) (%)	76 (83.5)	68 (73.9)	63 (68.5)	0.06
Number of people in the family^4^ (less than 4) (%)	57 (32.4)	62 (35.2)	63 (68.5)	0.7
Marital status (single) (%)	13 (14.3)	8 (8.7)	14 (15.2)	0.36
High economic status^5^ (%)	4 (4.4)	13 (14.1)	19 (20.7)^a^	0.005
Clinical history				
Disease history^6^ (%)	76 (83.5)	68 (73.9)	63 (68.5)	0.06
Diabetes (%)	50 (49.9)	37 (40.2)	23 (25)^a^^,^^b^	<0.001
Fatty liver (%)	26 (28.6)	16 (17.4)	12 (13)^a^	0.02
Taking medication^7^ (%)	79 (86.8)	85 (92.4)	80 (87)	0.39
Anti-inflammatory drug^8^ (%)	56 (61.5)	49 (53.3)	51 (55.4)	0.5
Nitroglycerin (%)	0	3 (3.3)	0	0.05
Fat reducing drug^9^ (%)	69 (75.8)	77 (83.7)	69 (75)	0.28
Anticoagulant^10^ (%)	23 (25.3)	36 (39.1)	13 (14.1)	0.001

BMI, body mass index and MET, metabolic equivalent of task.

1 Continuous variables are reported as mean ± SD. Categorical variables are reported as percentage.

2 *p*-values obtained from ANOVA and *χ*^2^ test for continuous and categorical variables, respectively.

3 Based on having university and non-university education.

4 Number of family members based on more than 4 people and less than 4 people.

5 Economic status based on foreign travel.

6 Including diabetes, fatty liver.

7 Including anti-inflammatory drug, nitroglycerin, fat-reducing drug, anticoagulant drug.

8 Including corticosteroid and non-steroidal.

9 Including statins, fibrates, ezetimibe, and niacin.

10 Including clopidogrel, dipyridamole, ticlopidine, warfarin, enoxaparin, rivaroxaban.

a Is significant compared to the first tertile.

b Is significant compared to the second tertile.

**Table 2:** Dietary intakes of study participants across tertile of DII (energy-adjusted).^1^

	T1 (*n* = 91)	T2 (*n* = 92)	T3 (*n* = 92)	*p*-value^2^
Food groups				
Whole grains (g/d)	167.30 ± 15.72	110.49 ± 11.73	129.09 ± 11.56^a^	<0.001
Refined grains (g/d)	192.68 ± 61.22	225.53 ± 59.52	398.92 ± 60.59^a^	0.04
Fruit (g/d)	571.30 ± 44.51	684.29 ± 38.92	687.93 ± 49.30	0.13
Vegetables (g/d)	285.38 ± 17.37	351.24 ± 22.36	386.92 ± 27.35^a^	0.02
Red and processed meat (g/d)	88.93 ± 6.45	92.81 ± 6.27	113.38 ± 6.36^a^	0.02
Dairy (g/d)	415.16 ± 30.93	346.60 ± 23.71	417.90 ± 29.48	0.15
Nuts (g/d)	12.16 ± 1.24	8.65 ± 1.21	7.38 ± 1.22^a^	0.02
Legumes (g/d)	34.82 ± 4.10	43.15 ± 3.99	56.45 ± 4.05^a^	0.001
Nutrients				
Energy intake (kcal/d)	1930 ± 420.45	2293.55 ± 485.20	2459 ± 455.25^a^	<0.001
Carbohydrates (% energy)	161.06 ± 24.61	152.86 ± 16.94	131.05 ± 17.28^a^^,^^b^	<0.001
Protein (% energy)	33.27 ± 5.06	36.71 ± 5.21	40.07 ± 6.00^a^^,^^b^	<0.001
Fat (% energy)	28.55 ± 9.75	31.12 ± 6.06	39.16 ± 7.03^a^^,^^b^	<0.001
SFA (g/d)	10.64 ± 0.39	11.40 ± 0.39	14.13 ± 0.39^a^^,^^b^	<0.001
PUFA (g/d)	5.04 ± 0.19	5.23 ± 0.196	6.91 ± 0.19^a^^,^^b^	<0.001
MUFA (g/d)	9.30 ± 0.33	10.49 ± 0.33	13.64 ± 0.32^a^^,^^b^	<0.001
Cholesterol (g/d)	88.59 ± 11.30	116.68 ± 11.23	225.38 ± 11.23^a^^,^^b^	<0.001
Omega 3 (g/d)	0.34 ± 0.02	0.37 ± 0.02	0.51 ± 0.02^a^^,^^b^	<0.001
Omega 6 (g/d)	0.02 ± 0.01	0.04 ± 0.01	0.05 ± 0.01^a^	0.001
Iron (mg/d)	11.55 ± 0.87	13.89 ± 0.87	19.98 ± 0.87^a^^,^^b^	<0.001
Magnesium (mg/d)	184.85 ± 3.62	191.82 ± 3.61	193.59 ± 3.61	0.19
Zinc (mg/d)	5.20 ± 0.11	5.80 ± 0.11	6.31 ± 0.11^a^^,^^b^	<0.001
Selenium (μg/d)	49.11 ± 1.47	44.77 ± 1.46	46.94 ± 1.46	0.11
Vitamin B1 (μg/d)	0.81 ± 0.02	0.72 ± 0.02	0.74 ± 0.02^a^	0.001
Vitamin B2 (mg/d)	0.75 ± 0.02	0.91 ± 0.02	1.05 ± 0.02^a^^,^^b^	<0.001
Vitamin B3 (mg/d)	9.48 ± 0.19	9.70 ± 0.19	9.68 ± 0.19	0.67
Vitamin B6 (mg/d)	0.88 ± 0.02	0.97 ± 0.02	1.04 ± 0.02^a^^,^^b^	<0.001
Vitamin B9 (μg/d)	209.23 ± 4.28	217.53 ± 4.25	226.76 ± 4.25^a^	0.02
Vitamin B12 (μg/d)	1.48 ± 0.11	2.10 ± 0.11	3.02 ± 0.11^a^^,^^b^	0.001
Vitamin C (mg/d)	61.06 ± 4.52	102.85 ± 4.50	94.37 ± 4.50^a^	<0.001
Vitamin D (μg/d)	0.55 ± 0.30	0.44 ± 0.29	0.42 ± 0.27^a^	0.04
Vitamin E (mg/d)	4.48 ± 0.16	4.96 ± 0.16	6.42 ± 0.16^a^^,^^b^	<0.001
Vitamin A (μg/d)	262.32 ± 15.90	421.27 ± 15.81	428.23 ± 15.81^a^	<0.001
Caffeine (g/d)	2.80 ± 0.23	2.75 ± 0.23	2.60 ± 0.23	0.83
Beta-carotene (μg/d)	2086.11 ± 187.27	3700.61 ± 186.28	3421.35 ± 186.25^a^	<0.001
Garlic (g/d)	0.81 ± 0.71	0.63 ± 0.70	0.62 ± 0.53^a^^,^^b^	0.11
Onion (g/d)	15.28 ± 10.92	19.30 ± 12.63	21.65 ± 12.87^a^	0.003
Pepper (g/d)	0.32 ± 0.07	0.16 ± 0.52	0.10 ± 0.24^a^	0.002
Tea (g/d)	0.77 ± 0.46	0.71 ± 0.39	0.60 ± 0.40^a^	0.02
DII	−5.55 ± 0.18	−0.592 ± 0.18	4.571 ± 0.18	<0.001

SFA, saturated fatty acids; MUFA, monounsaturated fatty acids; PUFA, polyunsaturated fatty acids.

1 Values are mean ± SD. Intakes of food groups and nutrients were adjusted for energy intake.

2 *p*-values obtained from ANOVA.

a Is significant compared to the first tertile.

b Is significant compared to the second tertile.

The raw and adjusted average severe CAD, lipid profile, and BP among the tertiles of DII are shown in Table 3. A significant difference was observed between the three levels of the DII in terms of the gensini score and the mean serum concentrations of q-CRP and total cholesterol, and this difference was also significant in the adjusted models. According to proximity tests, low adherence to the anti-inflammatory diet caused an increase in the gensini score (*p* < 0.001) and the mean concentrations of serum q-CRP (*p* < 0.001) and total cholesterol (*p* < 0.001). Also, those in higher tertiles of DII had higher mean systolic blood pressure than those in lower tertiles of DII (*p* = 0.04), which was not significant in the adjusted model. The crude and adjusted OR and 95% CI for severe CAD, high levels of lipid profile, high levels of q-CRP, and hypertension among the tertiles of DII are presented in Table 4. A direct and significant relationship was observed between the DII and severe CAD. People with the lowest adherence to anti-inflammatory diet had a 3.71 times higher chance of suffering from severe CAD than those with the highest adherence to anti-inflammatory (OR: 3.71; 95% CI: 1.97–6.98). This significance was also seen in the adjusted model, so that after adjusting the confounding variables, people with the lowest adherence to anti-inflammatory diet had a 6.09 times higher chance of suffering from severe CAD than those with the highest adherence to anti-inflammatory diet (OR: 6.09; 95% CI: 13.47–2.75). A direct and significant relationship was observed between the DII and increased q-CRP levels so that lower adherence to an anti-inflammatory diet increased the odds of q-CRP positivity by 2.11 times. However, this association disappeared after adjusting for confounding factors in the adjusted model (OR: 2.02; 95% CI: 0.86–4.73). There was a direct and significant relationship was found between lower adherence to an anti-inflammatory diet and hypercholesterolemia (OR of the third tertile compared to the first tertile: 2.73; 95% CI: 1.48–5.03), decreased HDL-cholesterol levels (OR of the tertile third compared to the first tertile: 3.77; 95% CI: 1.52–9.34) and hypertension (OR of the third tertile compared to the first tertile: 1.93; 95% CI: 3.49–1/06) in the raw model. After adjustment for the confounding factors, the relationship remained direct and significant. In the crude model, regarding the lower adherence to the anti-inflammatory diet, the chance of developing hypertriglyceridemia (OR: 2.02; 95% CI: 0.86–4-73) and LDL-C (OR: 2.02; 95% CI: 0.86–4.73) increased; however, this relationship was not statistically significant, and after adjusting for confounding factors, no significance was observed.

**Table 3:** Adjusted average values of gensini score, lipid profile, and BP among the tertiles of DII (energy-adjusted).^1^

		T1 (*n* = 91)	T2 (*n* = 92)	T3 (*n* = 92)	*p*-value^2^
Gensini score					
Crude model		36.40 ± 5.45	41.88 ± 5.42	47.62 ± 5.42^a^^,^^b^	0.002
Adjusted model		34.45 ± 5.29	52.55 ± 5.05	63.72 ± 5.18^a^^,^^b^	<0.001
Quantitative c-reactive protein (mg/L)					
Crude model		5.59 ± 0.37	4.58 ± 0.36	7.61 ± 0.37^a^^,^^b^	<0.001
Adjusted model		5.65 ± 0.37	4.56 ± 0.36	7.57 ± 0.36^a^^,^^b^	<0.001
Triglycerides (mg/dl)					
Crude model		175.87 ± 5.29	173.92 ± 5.26	190.67 ± 5.26	0.05
Adjusted model		175.98 ± 5.54	173.74 ± 5.29	190.74 ± 5.43	0.06
Total cholesterol (mg/dl)					
Crude model		188.27 ± 6.37	193.63 ± 6.33	229.41 ± 6.33^a^^,^^b^	<0.001
Adjusted model		190.71 ± 6.17	192.19 ± 6.17	228.42 ± 6.33^a^^,^^b^	<0.001
HDL-cholesterol (mg/dl)					
Crude model		59.53 ± 1.34	50.56 ± 1.33	56.27 ± 1.33	0.16
Adjusted model		59.78 ± 1.37	56.37 ± 1.31	56.15 ± 1.34	0.13
LDL-cholesterol (mg/dl)					
Crude model		143.19 ± 7.30	128.68 ± 7.26	148.28 ± 7.26	0.14
Adjusted model		142.9 ± 7.66	129.42 ± 7.32	147.82 ± 7.50	0.19
Systolic blood pressure (mmHg)					
Crude model		132.20 ± 1.45	131.7 ± 1.45	136.46 ± 1.45^b^	0.04
Adjusted model		132.73 ± 1.54	131.68 ± 1.47	136.05 ± 1.51	0.11
Diastolic blood pressure (mmHg)					
Crude model		79.67 ± 0.97	81.72 ± 0.97	82.96 ± 0.97	0.06
Adjusted model		79.79 ± 0.96	81.58 ± 0.92	82.99 ± 0.94	0.07

Adjusted model: Adjusted for energy, age, gender, body mass index, physical activity, Taking medication, medical history, number of family members, education.

1 Values are mean ± SE.

2 *p*-values obtained from ANCOVA.

a Is significant compared to the first tertile.

b Is significant compared to the second tertile.

**Table 4:** Adjusted odds ratio (OR) and 95% confidence interval (CI) for severe CAD, high levels of lipid profile, high levels of q-CRP, and hypertension among the tertiles of DII (energy-adjusted).^1^

		T1 (*n* = 91)	T2 (*n* = 92)	T3 (*n* = 92)	*p*-trend^2^
Severe coronary artery disease (gensini score > 20)					
Crude model		1.00 (Ref)	1.52 (0.85–2.71)	3.71 (1.97–6.98)	0.16
Adjusted model		1.00 (Ref)	2.11 (1.05–4.25)	6.09 (2.75–13.47)	<0.001
Quantitative c-reactive protein (>10 mg/L)					
Crude model		1.00 (Ref)	0.83 (0.37–1.87)	2.11 (1.03–4.29)	0.03
Adjusted model		1.00 (Ref)	0.91 (0.37–2.25)	2.02 (0.86–4.73)	0.07
Triglycerides (>150 mg/dl)					
Crude model		1.00 (Ref)	1.68 (0.87–3.27)	1.58 (0.82–3.05)	0.16
Adjusted model		1.00 (Ref)	1.85 (0.92–3.73)	1.69 (0.82–3.51)	0.15
Total cholesterol (>200 mg/dl)					
Crude model		1.00 (Ref)	1.47 (0.78–2.72)	2.73 (1.48–5.03)	0.001
Adjusted model		1.00 (Ref)	1.33 (0.68–2.60)	2.81 (1.41–5.61)	0.003
HDL-cholesterol (<40 mg/dl for men and <50 for women)					
Crude model		1.00 (Ref)	1.63 (0.60–4.4)	3.77 (1.52–9.34)	0.002
Adjusted model		1.00 (Ref)	2.03 (0.62–6.69)	6.68 (2.11–22.26)	0.001
LDL-cholesterol (>100 mg/dl)					
Crude model		1.00 (Ref)	0.71 (0.39–1.29)	1.28 (0.71–2.28)	0.40
Adjusted model		1.00 (Ref)	0.69 (0.36–1.32)	1.21 (0.63–2.31)	0.56
Hypertension (systolic blood pressure > 130 and diastolic blood pressure > 80)					
Crude model		1.00 (Ref)	0.89 (0.50–1.60)	1.93 (1.06–3.49)	0.033
Adjusted model		1.00 (Ref)	0.71 (0.36–1.42)	2.34 (1.08–5.06)	0.034

Adjusted model: Adjusted for energy, age, gender, body mass index, physical activity, Taking medication, medical history, number of family members, education.

1 All values are odds ratios and 95% confidence intervals obtained from Binary Logistic Regression.

2 *p*-trend was obtained by the use of DII tertiles as a continuous rather than categorical variable.

## Discussion

In the present study, a linear and direct relationship was observed between the DII and severe CAD. We also observed a significant difference between the DII tertiles in terms of gensini score. We found that a significant percentage of the participants were suffering from severe CAD (59.6%). Atherosclerosis is still the main cause of death with an increasing prevalence globally (34); thus, following anti-inflammatory regimens can positively affect the reduction of complications caused by blood clots.

Consistent with our study, other studies in the United States (35, 36), Australia (37), and Europe (38) showed that DII scores are positively associated with CAD risk. In a case–control study published in Jordan in 2019, a significant relationship was found between DII and the risk of CAD (18). In a prospective cohort study in Australia, the risk of CAD in men with a pro-inflammatory diet increased two times during the study (39). A randomized trial, PREDIMED, in Span showed that the risk of CAD in the fourth DII quartile increased by 73% compared to the first quartile (11). Also, in a case–control study conducted in northern Sweden, the risk of myocardial infarction in men with higher adherence to an inflammatory diet increased by 57% compared to people with low adherence (40). A meta-analysis, using data related to 14 eligible studies, examined the relationship between DII and the risk of CAD and its related mortality and it was found that the risk of CAD increased by 36% in people with higher adherence to the inflammatory diet (41). Another systematic review and meta-analysis conducted by Namazi et al. showed a positive and significant relationship between DII and the risk of CAD (42). In a cross-sectional study conducted in Iran, some components, such as nuts, showed an inverse and significant relationship with a decrease in the risk of the disease (21). However, our results are not consistent with some studies.

The contradictory results may be due to different food patterns, populations, sample sizes, and genetics. In the cross-sectional study conducted in Iran, no significant relationship was observed between red meat consumption and CAD. Patients with CAD reported more consumption of nuts and the use of fresh and cooked vegetables, dried fruits, animal oil cakes, fried potatoes, and some dairy products was correlated with CAD (20). Although several studies have been done on DII and its relationship with CAD, according to the researcher’s knowledge, no study has evaluated the relationship between DII and CAD severity using the gensini score in IRAN.

Several mechanisms have been proposed to explain the association between DII and vascular occlusion. Several theories have shown the consistent relationship between DII and the risk of developing CAD and its mortality; for example, the pro-inflammatory association of diet on increasing the level of cytokines, such as IL-1 and TNF-α, which causes the attraction and movement of inflammatory cells to the surface of the vascular endothelium (43) and induces the expression of cell adhesion molecules mediating leukocyte adhesion to the vascular endothelium (44).They also induce “messenger” cytokines, which increase the production of acute phase reactants, including CRP and serum amyloid A (SAA) through releasing into the systemic circulation (45). Inflammation in all stages of atherothrombosis is the main cause of about 80% of sudden cardiac deaths (SCD) (46). In previous studies, the DII score obtained from the FFQ was significantly associated with inflammatory biomarkers. Thus, higher DII scores (indicating a more inflammatory diet) have been directly related to IL-6, TNFα-R2, and CRP (15). IL-6 is the main pro-coagulant cytokine and can increase the concentration of fibrinogen, plasminogen activator inhibitor type 1 (47), and CRP, leading to an increase in pro-inflammatory and pro-coagulant responses (48). The basis of the relationship between CRP and atherosclerosis is the CRP’s potential to directly modulate the production of endothelium-derived vasoactive factors. Nitric oxide(NO) is the key factor in maintaining vascular tone and the central controller of cardiovascular homeostasis, which is derived from vascular endothelium (49). The reduced production or effect of NO through increased vascular contraction, leukocyte adhesion, platelet activation, oxidation, thrombosis, coagulation disorders, and vascular inflammation plays an essential role in the pathogenesis of the vascular atherosclerotic disease (50). Anti-inflammatory diet exert their effects on arterial blockage by reducing IL-6, TNFα-R2, and CRP levels (15). A number of studies have been shown that anti-inflammatory diet can modulate endothelium dependent vasodilation responses, endothelium-leukocyte interactions as well as balance between pro-and antithrombotic properties (51).

We also showed that lower adherence of an anti-inflammatory diet is related to an incremented risk of high blood pressure. The findings of the present study are confirmed by other studies indicating a positive relationship between the inflammatory potential of diet and hypertension (52, 53). Also, several prospective trials have associated increased inflammation with higher risks of hypertension (54). Inflammatory cytokines can strongly induce high blood pressure, which plays a role in regulating blood pressure due to the disruption of the renin-angiotensin system, vascular inflammation, and the reduction of NO production (55). The inflammation as well as the production of inflammatory cytokines activate the immune system and increase the expression of the angiotensinogen gene and angiotensin-converting enzyme (52), which ultimately causes the production of angiotensin 2, a strong constrictor, and increases blood pressure. On the other hand, inflammation and vascular damage can reduce the production of NO as a vasodilator, leading to high blood pressure (56).

Also, less following an anti-inflammatory diet was related to an increased risk of hypercholesterolemia and reduced HDL-C. A low-quality diet including excessive consumption of inflammatory food items increases lipogenesis (57, 58). A recently published prospective population-based study showed that a pro-inflammatory diet was associated with an increased risk of dyslipidemia (59). In a meta-analysis, higher levels of DII were associated with higher levels of TG and LDL-C in apparently healthy populations (60). The relationship between DII and increased TG and decreased HDL-C has also been reported (61). Therefore, the contradictory results may be due to different food patterns, populations, sample sizes, and genetics.

The present study had strengths and weaknesses. The severity of coronary artery disease was determined based on gensini’s score, and its validity has been confirmed. Nutritional intake was evaluated using valid questionnaires. In addition, the effects of several potential confounding factors were controlled in data analyses. However, some limitations should be considered in interpreting the findings. Due to the cross-sectional design of the study, we could not infer a causal relationship between the DII and CAD. More prospective studies should be conducted to confirm the causality of the associations. Although a validated FFQ was used to assess dietary intakes, recall bias may have influenced the findings. In addition, we did not have information about family history of CAD and 12 dietary items to calculate the DII score, which could affect the results.

This cross-sectional study showed a direct and linear relationship between the DII and the occurrence of severe CAD. Also, a significant difference was found between the DII tertiles in terms of gensini score. It is recommended people that in order to reduce the inflammatory potential of the diet, people should minimize the consumption of foods such as fast food, bread and pasta made with white flour, deep fried items such as french fries, fried chicken and donuts.

## Data availability statement

The raw data supporting the conclusions of this article will be made available by the authors, without undue reservation.

## Ethics statement

The studies involving humans were approved by Ethics Committee of Isfahan University of Medical Sciences. The studies were conducted in accordance with the local legislation and institutional requirements. The participants provided their written informed consent to participate in this study.

## Author contributions

ZD, MB, OS, AK, SS, and GA contributed in design, conception, data interpretation, data collection, approval of the final version of the manuscript, manuscript drafting, and agreed for all aspects of the work. All authors contributed to the article and approved the submitted version.

## Glossary

DII: Dietary inflammation index
FFQ: Food frequency questionnaire
OR: Odds ratios
95% CI: 95% Confidence interval
BMI: Body mass index
MUFA: Mono unsaturated fatty acid
PUFA: Poly unsaturated fatty acid
SFA: Saturated fatty acid
ANOVA: Analysis of variance
ANCOVA: Analysis of covariance
SPSS: Statistical package for the social sciences
SD: Standard deviation
CAD: Coronary artery disease
LDL-c: Low-density lipoprotein cholesterol
CVDs: Cardiovascular diseases
TG: Triglyceride
HDL-c: High-density lipoprotein cholesterol
BP: Blood pressure
IL-6: Interleukin-6
IL-1b: Interleukin-1 beta
TNFα-R2: Tumor necrosis factor receptor 2 alpha
SES: Socioeconomic status
IPAQ: International Physical Activity Questionnaire
Q-CRP: Quantitative C-reactive protein
N4: Nutritionist IV
AHEI: Alternative healthy eating index
SCD: Sudden cardiac deaths
Enos: Endothelial nitric oxide synthase
ET-1: Endothelin-1
ICAM-1: Intracellular adhesion molecule type 1
NO: Nitric oxide
MET: Metabolic Equivalent of Task
